# p53 in head and neck cancer: Functional consequences and environmental implications of *TP53 *mutations

**DOI:** 10.1186/1758-3284-2-36

**Published:** 2010-12-15

**Authors:** Jenni K Peltonen, Henni M Helppi, Paavo Pääkkö, Taina Turpeenniemi-Hujanen, Kirsi H Vähäkangas

**Affiliations:** 1 Institute of Clinical Medicine, Department of Oncology and Radiotherapy, University of Oulu, Oulu University Hospital, Oulu, Finland; 2 Institute of Biomedicine, Department of Pharmacology and Toxicology, University of Oulu, Oulu, Finland; 3 Institute of Diagnostic, Department of Pathology, University of Oulu, Oulu University Hospital, Oulu, Finland; 4 Faculty of Health Sciences, University of Eastern Finland, POB 1627, FI-70211 Kuopio, Finland

## Abstract

**Background:**

Although *TP53 *mutations in human tumours generally have been extensively studied, the significance of p53 in the aetiology of head and neck cancers is still incompletely characterized. In recent years, considerable interest has been focused on mutant forms of p53, the abnormal protein product of *TP53 *alleles with missense mutation that often accumulate in cancer cells.

**Methods:**

We compared the nature of *TP53 *mutations in primary 46 head and neck squamous cell carcinomas (HNSCC) analyzed by PCR-SSCP and sequencing, immunohistochemistry, and using structural information available at IARC p53 database.

**Results:**

Sequencing confirmed 36 *TP53 *mutations in 23 tumours of the 39 mutations in 26 tumours found by PCR-SSCP. Only half (17) putatively affect the function of p53 protein. Of these 8 were in the L2 domain, three affected the LSH motif and three the L3 domain. Three were in other domains. Codon 259 (GAC > GAA) which is a very rare mutation was found in 4 samples in our study. There were indications of p53 aberrations being associated with the combined effect of smoking, alcohol and work history. Patients with a negative family history of cancer had more often *TP53 *mutations than patients with a positive family history (71% vs. 46%).

**Conclusions:**

Our study contributes to the knowledge of cumulative chemical exposure and p53 aberrations in head and neck cancer, an area where literature is scarce.

## Introduction

Carcinomas of the head and neck are among the most common types of cancer [1] and as such represent a major health problem. Although it is difficult to distinguish the effects and risks of individual carcinogens from all other exposures, it is clear that head and neck squamous cell carcinoma (HNSCC) is epidemiologically strongly associated with alcohol consumption and exposure to tobacco smoke [2]. The probability of developing the cancer increases with the amount of tobacco and alcohol consumed [3,4]. Associations between head and neck cancer risk and exposures to other environmental and occupational factors have also been proposed [5]. Putative occupational risk factors include nickel refining, woodworking, and exposure to textile fibres. Moreover most studies suggest that oral cancer patients have a history of diet low in fruit and vegetables [6]. In addition, human papillomavirus (HPV) infection has been associated with some HNSCC subgroups, mostly cancer in oropharynx [7,8]. A synergistic effect between exposures is likely, because synergism has been demonstrated between smoking and radon or asbestos in lung cancer and oesophageal cancers [4,9].

Aberrations of p53 are the most frequent molecular events in human cancers. The *TP53 *tumour suppressor gene in chromosome 17p13.1 encodes the p53 protein involved in many key events in the cell like regulation of cell cycle and glucose metabolism in cancer cells, DNA-repair, apoptosis, and senescence and induced by various stress signals, including DNA-damage and inflammation [10,11]. In both mice and humans, germ line mutations in *TP*53 result in a strong predisposition to cancer [12]. Indeed, Gadea and co workers (2007) showed that a loss of wild-type p53 function was enough by itself to confer an increased migratory capacity to cells [13]. It has been shown that there are less *TP53 *mutations in the setting of HPV infection [14,15]. The biological basis for this is provided by the fact that the HPV E6 oncoprotein specifically inactivates wild-type p53. In this way the high-risk HPV E6-mediated degradation of the p53 protein is probably an alternative pathway for a "classical" mutation to knock-out the p53 regulated pathways [15,16]. Analysis of *TP53 *mutational patterns has shown its usefulness in at least two main areas [17,18]. Firstly, knowledge of the position of mutations has helped to better understand the functions of various domains of the p53 protein and their involvement in mediating the suppressive functions that are inactivated in cancer. Secondly, it has been shown that the patterns of mutations may vary according to the nature of etiological agents implicating the use of *TP53 *mutation spectrum as a biomarker of environmental aetiology. Most of *TP53 *mutations described in the IARC *TP53 *mutation database affect exons 5-8, which constitute the site-specific, DNA-binding domain [19]. This region encodes for residues 130-286, also the most important region for folding and stabilization of the tertiary structure of p53 protein. Less than 2% of the mutations are found in the N- and C-terminal regulatory domains. The crystal structure of the core domain, solved in 1994, provides a template for understanding the nature of mutant p53 [20]. The structure contains a β-sandwich scaffold and a DNA-binding surface, including a loop-sheet-helix (LSH) motif and two loops (L2 and L3) tethered by a single zinc atom. Different mutations have very different consequences for the function of p53 protein. However, mostly mutation frequencies in tumours have been reported and less attention has been paid to the connection of functional state of the mutated p53 with clinical and environmental aspects of cancer. Most of the *TP53 *mutations in human cancers are missense mutations [17], that can either cause a loss of tumour suppressor function (LOF) or, in some cases, a gain of oncogenic function (GOF) [21,22]. In addition to various degree of LOF, some mutant proteins inhibit the functions of the wt allele by a dominant-negative effect [19]. Recent studies have been carried out in an attempt to provide an explanation for the structural effects of most disease-related *TP53 *mutations [23,24] and functional impact of *TP53 *mutations [25,26].

In this study, we have analyzed p53 aberrations in primary head and neck cancer patients with information of their chemical exposures. Although the association between smoking and alcohol with head and neck cancers is well-known and quite strong [2], and it is clear that p53 aberrations in general are important in human cancers, it is not known whether p53 aberrations are associated with environmental exposures in head and neck cancers. The 46 head and neck cancers analyzed in this paper provide a significant addition the data in IARC *TP53 *mutation database, especially due to the known environmental exposures.

## Materials and methods

### Patients and tumours

The study population consisted of North Finnish patients diagnosed with a primary head and neck squamous cell carcinoma in the University Hospital of Oulu, Finland between the years 1994 and 1996. The patients were recruited to the study when entering to hospital. Details of the cases are given in Table 1. In each case, a questionnaire was filled about smoking, alcohol consumption and work place with a possibility of exposure to chemicals at work, as well as the family history. Questions were asked during the first contact with the cancer clinic by an experienced doctor or nurse. An exposure index (Table 2) was calculated using the data from the structured questionnaire on lifestyle and work history, as well as on the exposure to chemicals. The maximum rating was eight points including 0-3 points from tobacco exposure, 0-3 points from alcohol consumption and 0-2 points from possible exposure to chemicals and/or dust.

**Table 1:** Clinicopathological variables and p53 status in patients of head and neck carcinoma

Patient characteristics	n	*TP53 *mutation n/n(%)	p53 ihc positive n/n(%)
All patients	46	26/46 (56.5%)	24/46 (52.2%)

Sex			

Male	31	18/31 (58.1%)	19/31 (61.3%)

Female	15	8/5 (53.3%)	5/15 (33.3%)

Age, years			

≤ 39	3	3/3 (100.0%)	2/3 (66.7%)

40-65	23	13/23 (56.5%)	10/23 (43.5%)

≥ 66	20	10/20 (50.0%)	12/20 (60.0%)

Anatomical diagnosis			

Oral cavity	14	9/14 (64.3%)	4/14 (28.6%)

Larynx	24	13/24 (54.2%)	19/24 (79.2%)

Pharynx	6	4/6 (66.7%)	1/6 (16.7%)

Others	2	0/2 (0%)	0/2 (0%)

Grade			

Grade 1	10	5/10 (50.0%)	5/10 (50.0%)

Grade 2	29	17/29 (58.6%)	14/29 (48.3%)

Grade 3	7	4/7 (57.1%)	5/7 (71.4%)

TNM classification			

T_1-2_	29	14/29 (48.3%)	17/29 (58.6%)

T_3-4_	17	12/17 (70.6%)	7/17 (41.2%)

N_0_	23	14/23 (60.9%)	13/23 (56.5%)

N_+_	23	12/23 (52.2%)	11/23 (47.8%)

Stage			

I	6	4/6 (66.7%)	3/6 (50.0%)

II	9	4/9 (44.4%)	7/9 (77.8%)

III	20	11/20 (55.0%)	10/20 (50.0%)

IV	11	7/11 (63.6%)	4/11 (36.4%)

**Table 2:** The points of the exposure index

Exposure points	Tobacco exposure description	Alcohol exposure description	Chemical/dust exposure description
0	Non-smoker	No alcohol consumption	No exposure
1	Pack years 1-10	Occasionally (1-2 times/month)	
2	Pack years 11-45	Weekly (1-2 times/week)	Exposure to a chemical and/or dust
3	Pack years over 45	Daily (heavy drinking)	

### Ethical aspect

The study design was approved by the local Research Ethics Committee of the Medical Faculty and University Hospital at the University Of Oulu, Finland (14.3.1994) and a written informed consent was obtained from all patients entering the study, after both oral and written information was given to the patients about the study. Patients were interviewed by hospital personnel (a doctor or a nurse) and the coded data was stored in a safe place by the researches. The study did not interfere with the clinical treatment of the patients.

### TP53 mutation analysis strategy

Mutations in exons 5-8 of the *TP53 *gene were analyzed by a temperature-controlled non-radioactive single-strand conformation polymorphism (SSCP) analysis [27,28]. A sample was judged to be positive for a *TP53 *mutation in SSCP only if two independent amplified PCR products contained similar shifted band patterns. The types of the *TP53 *mutations were further analyzed by semi-automatic sequencing.

### Analysis of TP53 mutations with single-strand conformation polymorphism (SSCP)

Exons 5-8 of the *TP53 *gene were separately amplified by PCR using two sets of intron primers, the second set internal to the first (nested primers) [29]. Dynazyme DNA polymerase and the corresponding buffer (Finnzymes, Espoo, Finland) were used in the polymerase chain reaction (PCR) with other reagents and under the reaction conditions described previously [28]. To check for possible contamination, the first and the last reactions in each PCR series were controls with no template in the reaction. If a band appeared indicating contamination, the whole series of concurrent PCR reactions was discarded. The amplified products were purified by agarose gel electrophoresis, as described earlier [28]. In this non-radioactive SSCP method the use of two running temperatures in combination with other optimized conditions ensures 98% efficiency in mutation detection within the studied exons [27]. Pharmacia PhastSystem^® ^semi-dry electrophoresis equipment was used for SSCP, as described earlier [28]. Two different temperatures (4°C and 20°C) were used to obtain good efficiency. Both negative and positive controls were included in each run to ensure the quality of the run. As a negative control, gel-purified, amplified normal *TP53 *DNA was used. The controls were confirmed to be negative by identical band patterns compared to former controls, and sequenced to be wild-type. As a positive control, DNA was amplified using artificially mutated primers [27]. The gels were stained with silver staining kit (Pharmacia Biotech, Finland) according to the instructions from the manufacture.

### Sequencing of *TP53 *gene

Once a mutation was detected by the presence of similar band shifts in SSCP from two independent PCR, the PCR amplified samples were sequenced with ABI PRISM 3100 sequencer and BigDye Terminator Sequencing Kit (Applied Biosystems, Foster City, CA).

### Analysis of the effect of *TP53 *mutation

IARC *TP53 *mutation database (R13, released in November 2008) was searched for the mutations found in this study [19]. The mutation validation tools were used to check the mutation data for base substitutions in the coding sequence of *TP53*. The following information was used: the precise description of the mutation event at the DNA and protein level, the observed (in experimental cell assays [30] or predicted (by amino-acid conservation rules or structural analysis) functional impact of the mutation, and the number of times it has been reported as a somatic or germ line mutation in the IARC *TP53 *database. A combination of standard structural criteria as described by Martin *et al*. (2002) was also used [24]. The following changes were considered to have probable functional consequences: changes in amino acids involved in hydrogen bonding (as already described by Baker and Hubbard 1984) [31], substitutions with amino acids too large to fit in the place (residue clashes), mutations to proline (cyclic side chain in proline creates a stricter backbone than other amino acids), mutations substituting glycine (able to adopt conformations sterically hindered for other amino acids), or substitutions leading to changes in direct contact with DNA or zinc binding [24].

### p53 immunohistochemistry

Paraffin embedded sections (4 μm) were stained using the avidin-biotin-immunoperoxidase technique. Dewaxing (Histo-Clear^®^, National Diagnostic, Atlanta, GA, USA) and blocking of endogenous peroxidase and non-specific binding were carried out first. Mouse monoclonal antibody (DO-7, 1:300, Novocastra Laboratories Ltd., Newcastle upon Tyne, UK) for p53 was used as the primary antibody. The antibody recognizes both wild type and mutant forms of human p53 and the epitope is located between the amino acid residue 19 and 26. For staining the Histostain-bulk kit^® ^(Zymed, San Francisco, CA, USA) was used. Biotinylated antimouse IgG was used as the secondary antibody and peroxidase was introduced as a streptavidin conjugate. The antibody reaction was visualised by using a fresh substrate solution containing aminoethyl carbazol (AEC-kit^®^, Zymed, San Francisco, CA, USA). The sections were counterstained with hematoxylin, dehydrated and mounted in glycerol-vinyl-alcohol (GVA mount^®^; Zymed). For the negative controls the primary antibody for p53 was replaced with mouse non-immuno IgG and each set of staining always included a separate known positive control. The slides were analysed separately by two independent observers blinded from the clinical data. The immunoreactivity in the malignant cells in each section was graded according to the number of positively staining nuclei: < 1% nuclei with a positive reaction as a negative, >1≤6% +, >6% ≤10% as ++ 11% ≤40% as +++ and > 40% as ++++.

### Statistical analysis

The correlations of gender, age, primary anatomical site and exposure data were analyzed separately according to the *TP53 *gene mutations and p53 immunoreactivity. The statistical significance of these correlations was determined with the Fisher's exact test. Probability values of less than 0.05 were considered to be statistically significant. All statistical analyses were performed using the SPSS software system (SPSS for Windows, version 16.0, Chicago, IL).

## Results

### Mutations in the *TP53 *gene in head and neck tumours

Judging by SSCP the *TP53 *gene was mutated in a total of 26 primary tumours (57%) in the 46 HNSCC patients with altogether 39 *TP53 *mutations. Sequence analysis for the exact site and nature of the genetic alterations was possible in 23 tumour samples. Eleven tumour samples (11/26, 42%) were found to harbour multiple *TP53 *mutations. In two cases 3 mutations were found in the same tumour and in nine cases two (Table 3). There was only a small difference in the prevalence of mutations between different tumour sites (Table 4). The majority of the mutations were missense mutations (30/36, 83%). Only one of the mutations was a nonsense mutation and two were silent. Transversions (17/31, 55%) were more frequent than transitions (14/31, 45%). The two silent mutations found in codon 170 were similar (AC**G **> AC**A**) and both were found in association with a similar codon 171 missense mutation (GA**G **> GA**C**). One of these combination mutations was found in a larynx tumour and the other in oral cavity cancer. Furthermore, codon 259 was similarly mutated in 4 samples (GA**C **> GA**A**).

**Table 3:** Individual mutations and p53 protein function

Sample	Exon(s)	Muta-ted codon	Mutation by sequencing	AA change	Change in properties	Structural motif ^a^	Protein function
H&N 12	5 5 7	130 139 225	CTC>ATC AAG>TGG GTT > GCT	Leu > Ile Lys > Trp Val > Ala	no change +charged > aromatic hydrophobic > polar	LSH L L	NF ND F
H&N 18	5	155	ACC > AGCTGC	extra Cys	small	L	ND
H&N 19	5 7	157 258	GTC>TTC GAA > AAA	Val > Phe Glu > Lys	hydrophobic, small >aromatic, big -charge, acidic > +charge	S4 S9	NF^b^ NF^b^
H&N 20	7 8	243 297	ATG>CTG CAC > TAC	Met > Leu His > Tyr	Leu aliphatic no change	L3 (D)	NF^b^ ND
H&N 31	5	171	GAG > GAC	Glu > Asp	big >small	L2 (S)	F
H&N 29	7	259	GAC > GAA	Asp > Glu	small > big	L	NF^b^
H&N 32	5 8	171 271	GAG>GAC GAG > TAG	Glu > Asp Glu > stop	big >small	L2 (S) S10	F ND
H&N 28	8	14496	16 bases deletion	Frameshift mutation			ND
H&N 3	5 7	159 254	GCC >ACC ATC > GTC	Ala >Thr Ile > Val	no change big > small	S4 S9	F^b^ F^b^
H&N 16	5 7	130 245	CTC>TTC GGC > GAC	Leu > Phe Gly > Asp	aliphatic > aromatic polar > acidic	LSH L3 (D)	NF^b^ NF^b^
H&N 4	5 8	184 283	-1G CGC > CCC	Frameshift Arg >Pro	+ charged > polar	H2 (LSH) (D)	NF^b, d^
H&N 58	7	259	GAC > GAA	Asp > Glu	small > big	L	NF^b^
H&N 53	5	172	GTT > GCT	Val > Ala	hydrophobic > polar	L2 (S)	F^b^
H&N 64	7	238	TGT > TCT	Cys > Ser	hydrophobic > polar	L3 (D)	NF^b^
H&N 63	6	189	GCC > GTC	Ala > Val	polar > hydrophobic	L2 (S)	F^b^
H&N 51	7	259	GAC > GAA	Asp > Glu	small > big	L	NF^b^
H&N 54	7	259-260	GA**CT**CC > GA**TC**CC	Asp, Ser > Asp, Pro	no change	L	NF^d^
H&N 56	8	275	TGT > TAT	Cys > Tyr	small > big, aromatic	LSH	NF^b^
H&N 46	6	217	GTG > GCG	Val > Ala	hydrophobic > polar	S7	F^b^
H&N 43	5	155 175	ACC>TCC CGC > CAC	Thr > Ser Arg > His	no change + charged > big, aromatic	L L2 (S)	F^b^ NF ^b, c^
H&N 61	5	171	GAG > GAC	Glu > Asp	big >small	L2 (S)	F
**Sample**	**Exon(s)**	**Muta-ted codon**	**Mutation by sequencing**	**AA change**	**Change in properties**	**Structural motif ^a^**	**Protein function**
H&N 60	5 7	172 259	GTT>CTT GAC > GAA	Val > Leu Glu > Asp	small > big big >small	L2 (S) L	F NF^b^
H&N 69	5 6	148 221	GAT>GAG GAG > GAC	Asp > Glu Glu > Asp	small > big big >small	L L	F^b^ F^b^

a) D = mutation at the DNA contact site, S = structural mutation (according to the IARC classification)

b) NF = non-functional protein according to functional assays by Kato *et al*. 2003 ^30 ^, F = functional protein according to functional assays by Kato *et al*. 2003 ^30^, c) gain of function, d) mutations changing proline may also lead to an incorrectly folded protein due to the cyclic side chain of proline, ND = not determinable

**Table 4:** Summary of mutations

Anatomic site	Number of cases	Total number (%) of mutated cases	Total number of *TP53 *mutations	*TP53 *mutation frequency in different exons			
				Exon 5	Exon 6	Exon 7	Exon 8
**larynx**	24	13/24 (54%)	19	9/19 (47%)	3/19 (16%)	5/19 (26%)	2/19 (10.5%)
**pharynx**	6	4/6 (67%)	5	2/5 (40%)	0	3/5 (60%)	1/5 (20%)
**oral cavity**	14	9/14 (64%)	14	6/14 (43%)	0	4/14 (29%)	4/14 (29%)
**nose & sinuses**	2	0	0	0	0	0	0
**all sites**	46	26/46 (56.5%)	39	17/39 (43%)	3/39 (8%)	12/39 (31%)	7/39 (18%)

### Correlation of IHC for p53 protein with *TP53 *mutation status

**Figure 1:**
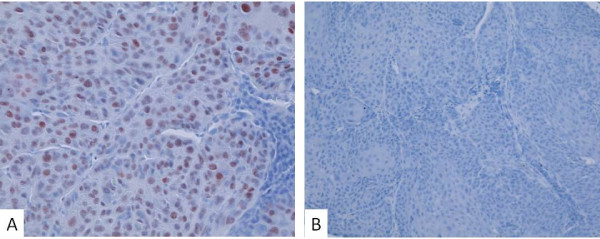
**Immunohistochemical staining of p53 immunoreactive protein (A, B)**. Nuclear immunostaining of p53 in head and neck squamous cell carcinoma. The immunostaining procedure was performed as described using an anti-p53 monoclonal antibody. A) Tumour with a very extensive (++++) immunostaining for p53 B) Tumour with a negative immunostaining for p53.

The p53 protein was analyzed in sections of the tumour samples by immunohistochemistry. In positive cases, the immunoreactive protein was prominent only in cancer cells and localized in cancer cell nuclei (Figure 1). Of the 46 primary HNSCC tumours, 24 (52%) showed a positive staining for the p53 protein. In 10 (22%) of the cases, the staining was extensive or very extensive (+++/++++), whereas 10 out of the 46 (22%) cases showed weak positivity (+) for p53 and in four cases (9%) the staining result was moderate (++). In cases where the tumour staining was very extensive or extensive for p53, 8 out of 10 cases (80%) contained also a *TP53 *mutation, while 17 out of 32 cases (53%) presenting with a negative or weak p53 staining contained a *TP53 *mutation in the tumour. The association between p53 immunohistochemical staining and the *TP53 *mutation status was not, however, statistically significant (P = 0.16, Fisher's exact test). There was no correlation between the type of the mutation and the positivity of p53 immunostaining.

### Effect of *TP53 *mutations on the p53 protein structure and function

According to the functional and structural domains of p53, as described in the IARC *TP53 *mutation database, the mutations could be classified as follows: 22% (8/36) of the mutations affect the L2 domain (between codons 164 and 194), which is needed for the correct folding and stabilization of the central part of the protein, 11% (4/36) affect the LSH (loop-sheet-helix) motif (codons 119-135 and 272-287), and 8% (3/36) affect the L3 domain (between codons 237-250), directly involved in the interaction between the protein and DNA (Table 3). According to the IARC database and based on experimental data, four of the missense mutations lead to non-functional proteins (Cys238Ser, Gly245Asp, Glu258Lys, Arg283Pro) (see Table 3). According to the predicted structure (by amino-acid conservation rules or structural analysis), several of the found mutations probably have functional impact leading to a non-functional protein (Leu130Ile, Leu130Phe, Thr155Ser, Val157Phe, Val172Ala, Arg175His, Met243Leu, Asp259Glu, Cys275Tyr). *TP53 *mutations leading to non-functional protein were more common in LSH and L3 motifs than in L2 motif (not statistically significant, data not shown).

### Comparison of *TP53 *gene and p53 protein alterations with patients characteristics and exposure data

Patients with a negative family history of cancer had a *TP53 *mutation in 71% of the cases, while patients with a positive family history (at least two cases of cancer in first-degree relatives) had a *TP53 *mutation in only 46% of the cases. *TP53 *mutations were more common (although not statistically significantly; p = 0.330) in tumours of younger patients (Table 1). All three patients under 39 years, who all had a tumour in the oral cavity, had a *TP53 *mutation. Otherwise *TP53 *mutation status was not associated with patient characteristics (Table 1). Interestingly, however, males had a p53 positive tumour more often than females (see Table 1 not statistically significant). When taking into account tobacco and alcohol consumption, the female patients had lower mean exposure (1.76 exposure index, see Table 2), than the males (3.36, p = 0.040). No statistically significant difference between the sexes was found, however, when the overall exposure was considered (p = 0.090). Another interesting finding was that p53 overexpression was more (although not statistically significantly) prevalent in laryngeal tumours than in other anatomical sites (Table 1). All tumours from patients with >45 pack years of smoking were p53 positive in ihc compared to those with 0 pack years (p = 0.021) (Table 5). No statistically significant association between p53 alterations and exposure to alcohol consumption was found. However, when all the exposures were taken into account, a positive result (p53 immunohistochemistry, *TP53 *mutation) seem to be more prevalent in those with a higher exposure index (Table 6). We also noticed that tobacco and alcohol exposures were significantly higher in laryngeal tumours than in oral cavity tumours (p < 0.005). No association was found between a high exposure index and the status with a non-functional protein. Unfortunately, in 37.5% of cases evaluated for functional consequences of the *TP53 *mutations it was not possible to [19] and that there is still minimal information about chemicals and *TP53 *in head and neck cancer [18] this calculate the exposure index due to partly lacking exposure data.

**Table 5:** Association of p53 aberrations and packyears of smoking

Packyears	Positive p53 IHC patient number in group	*TP53 *mutation patient number in group
0	2/8 (25%)	5/8 (63%)
1-10	4/7 (57%)	5/7 (71%)
11-45	11/24 (46%)	12/24 (50%)
Over 45	7/7 (100%)	4/7 (57%)

**Table 6:** Association of p53 aberrations and exposure index

Exposure Index^a^	Positive p53 IHC (number of patients)		*TP53 *mutation (number of patients)	
0	1/2		2/2	
1	-	3/8 (37.5%)	-	3/8 (37.5%)
2	2/6		1/6	
3	1/4		3/4	
4	4/9	10/20 (50%)	3/9	9/20 (45%)
5	5/7		3/7	
6	4/5		5/5	
7	3/4	8/10 (80%)	2/4	8/10 (80%)
8	1/1		1/1	

^a ^tobacco, alcohol, chemical/dust exposure, see table 2

## Discussion

The most interesting finding was that we found the same mutation (Asp259Glu most probably leading to a non-functional p53 protein) in four individuals who all had chemical exposure: tobacco and alcohol, and in three cases documented work exposure to chemicals including pesticides, oil and asbestos. In the fourth case the information of work exposure was missing. Although smoking and alcohol in head and neck cancer have been linked with *TP53 *mutations before [32-36] work exposure has not been included in earlier papers. Furthermore, considering the fact that this mutation has been described only in five cases before in IARC database is certainly an implication to follow-up. Including our cases, out of total of nine Asp259Glu mutations four have been in larynx tumours, which may represent a preferential site for this mutation. Both of our larynx cancer patients with this mutation tumour had a high exposure index (6 and 8) and none of the patients presented a positive family history for cancer. This may justify further studies of *TP*53 Asp259Glu mutation as a marker of environmental exposure in larynx tumours.

The *TP53 *mutation frequency in this study is in line with the one reported by IARC in head and neck squamous cell carcinomas (57% in this study; 47.5% in IARC *TP53 *mutation database [19]. According to the IARC *TP53 *mutation database, mutations in head and neck cancers occur frequently in codons 238-248, which is a hotspot region. In our material this region was underrepresented with only 3/26 mutations. Among this series, 11/26 (42%) tumours contained multiple *TP53 *mutations. Although multiple *TP53 *mutations have earlier been described in the literature in HNSCCs [37-40], they are not as commonly reported as tumours with a single *TP53 *mutation. Our study is too small to pursue multiple mutations in connection with other parameters.

Altogether 13 tumours with *TP53 *mutations in our series probably harbour a non-functional protein for various reasons. For instance, zinc is essential for the function of p53, because p53 does not adopt the correct conformation in the absence of zinc [41,42]. Thus mutations in the residues involved in the interaction with zinc, like Cys238Ser in this study, will result in a non-functional p53. According to recent data a mutation that alters the stability of the protein (structural mutants; 7 tumours in our series) is more likely to disrupt all functions of the protein, whereas a mutation within a contact residue (contact mutants; 4 cases in our series) will probably be more selective in affecting the transcriptional activity of p53 [43,44]. Both classes of mutant p53 proteins commonly accumulate to high levels in tumour cells and are defective for wild-type in p53 functions [43]. It remains to be tested whether any relation of the functional effects of the mutations to exposure types or total exposure exists. Our series is small and the fact that we did not find such correlations does not rule them out.

In accordance with Koch and co workers (1995) our study implicated young age to associate with a higher *TP53 *mutation frequency in the tumours [45]. Interestingly, all the three patients under 39 had a squamous cell cancer of the oral cavity and all these tumours harboured a *TP53 *mutation. Petitjean *et al*. (2007a), on the basis of the IARC *TP53 *mutation database, reported that the mean age at onset in carriers of a *TP53 *mutation leading to a functional protein was higher than the age of patients with a non-functional protein [25]. Thus, the penetrance of a mutation may be related to its degree of loss of transcriptional activity, which is not surprising. Similar implications have been described on basis of p53 protein expression. De Paula and co-workers (2009), who evaluated 724 primary HNSCC in young (under 45 years) and older (46-92 years) patients, reported a significantly higher p53 expression (p < 0.05) and a higher incidence of oral cavity tumours in younger patients [46]. On the other hand, Regezi *et al*. (1999), who evaluated and compared the expression of the cell cycle proteins p53, p21, Rb and MDM2 in tongue cancer patients aged 35 and younger and those aged 75 or older, reported equivalent p53 mutant protein expression [47]. The possible association with age in different head and neck cancers still requires confirmation in larger patient materials.

Positive findings in p53 immunohistochemistry have especially earlier, been interpreted as indicating inactivation of the *TP*53 gene on the basis of the knowledge that the half-life of the wild-type protein is too short to permit detection, whereas the mutant protein is stable [48]. However, the *TP53 *gene may also harbour mutations that do not result in its stabilization, or deletions that inhibit transcription altogether. Alternatively, p53 function may be inhibited by epigenetic events, such as enhancing its degradation or by interference with proteins controlling its transcriptional activity [49]. Furthermore, p53 protein may be induced in cells by DNA-damaging chemical exposure (for reviews see [50,51]) through posttranslational modifications [52]. In agreement with previous studies [48,53], we found that p53 overexpression was a common event in HNSCC. In newest papers immunohistochemically positive cases associate with *TP53 *mutations in head and neck cancers [48,54,55] and our paper does not contradict this.

In the present study, we found that a positive p53 immunohistochemistry was more common among heavy smokers than among non-smokers, as reported earlier [54,56]. However, we did not find a correlation between the amount of tobacco consumed and the frequency of *TP53 *mutations. Vähäkangas and co workers (2001) noticed that in lung cancer *TP53 *mutations occur more commonly in smokers and ex-smokers than in never-smokers [57]. Other reports pertinent to tobacco exposure, using various methods to detect *TP53 *mutations, have given conflicting results. Studies showing a positive association between *TP53 *mutation and tobacco smoke in patients with head and neck carcinoma are, however, more numerous e.g. [32-36] than studies with no association e.g. [38,58,59]. Unfortunately, information on alcohol consumption was lacking in eight patients in our material. Interestingly, we also found that the tumours from patients with a negative family history for cancer contained *TP53 *mutations more often than tumours from patients with a positive a family history. Considering the age correlation and family history data, our results may be interpreted as supporting the environmental aetiology of the *TP53 *mutations.

We noticed that tobacco and alcohol exposures were statistically significantly higher in laryngeal tumours than in oral cavity tumours and p53 overexpression was more prevalent in laryngeal tumours than in other anatomical sites. Recently De Paula and co-workers (2009) in a large material of over 700 patients noted a correlation between p53 immunohistochemically positive tumours and anatomical site [46]. The exposure to chemical carcinogens e.g. in smoke may not be even in different locations or the sensitivity of locations may vary. Another level of variation is the inter-individual susceptibility according to genetic factors. A small proportion of individuals exposed to potential carcinogens might develop the disease and intrinsic susceptibility to environmental exposure most probably plays a role also in head and neck cancer see e.g. [60,61]. Furthermore, in our series females with a head and neck cancer had less exposure than males, which supports the reported higher susceptibility of women to carcinogens like cigarette smoke [62].

## Conclusions

Our study shows implications of p53 aberrations being associated with the environmental exposure in head and neck cancer. Regardless of how the data are looked at, a trend for a higher frequency of p53 alterations remains among those with higher exposure. In accordance with earlier literature (see e.g. Vähäkangas 2003), our results thus justify further studies of p53 alterations as a biomarker of environmental exposure in head and neck cancers. Especially, the mutation Asp259Glu (GA**C **> GA**A**) most probably leading to a non-functional p53 protein, which was found in four tumours in this series, may justify further studies as a marker of environmental exposure in larynx tumours.

## Competing interests

The authors declare that they have no competing interests.

## Authors' contributions

JKP carried out mutation analysis, analyzed the mutation data and wrote the paper with KHV. HMH carried out immunohistochemical analysis and statistical analysis, wrote relevant methods parts and commented the manuscript. PP provided pathology expertise and commented the manuscript, T-TH took part in designing the study, was responsible for identifying and interviewing the patients and commented the manuscript, KHV designed and supervised the study, took part in the analysis of data, wrote the manuscript with JKP and edited the manuscript. All authors approved the final manuscript.
